# Bis[*N*′-(9*H*-fluoren-9-yl­idene)benzohydrazidato-κ^2^ *N*′,*O*]copper(II)

**DOI:** 10.1107/S1600536811038931

**Published:** 2011-10-05

**Authors:** Yan-Ling Guo, Wei Dou, Wei-Sheng Liu, Hong-Rui Zhang

**Affiliations:** a Nuclear Science and Technology, Lanzhou University, Lanzhou 730000, People’s Republic of China; b College of Chemistry and Chemical Engineering, Lanzhou University, Lanzhou 730000, People’s Republic of China

## Abstract

In the title complex, [Cu(C_20_H_13_N_2_O)_2_], the Cu^II^ ion is tetra-coordinated by an N_2_O_2_ set of two ligands in a distorted recta­ngular-planar geometry. The dihedral angle between the two coordinated five-membered metalla rings is 37.5 (3)°. The mol­ecular configuration is stabilized by two C—H⋯O and two C—H⋯N intra­molecular hydrogen bonds. The crystal packing is dominated by van der Waals inter­actions. Three atoms of the phenyl ring of the benzohydrazidate moiety are disordered over two sets of sites in a 0.625 (18):0.375 (18) ratio.

## Related literature

For general backgound to the biological and pharmacological activity of aroylhydrazones, see Ranford *et al.* (1998 ▶); Zhong *et al.* (2007 ▶); Wang *et al.* (2009 ▶); Li *et al.* (2010 ▶). For Schiff base coordination modes, see: El-Sherif (2009 ▶); Yang *et al.* (2006 ▶); Carcelli *et al.* (1995 ▶).
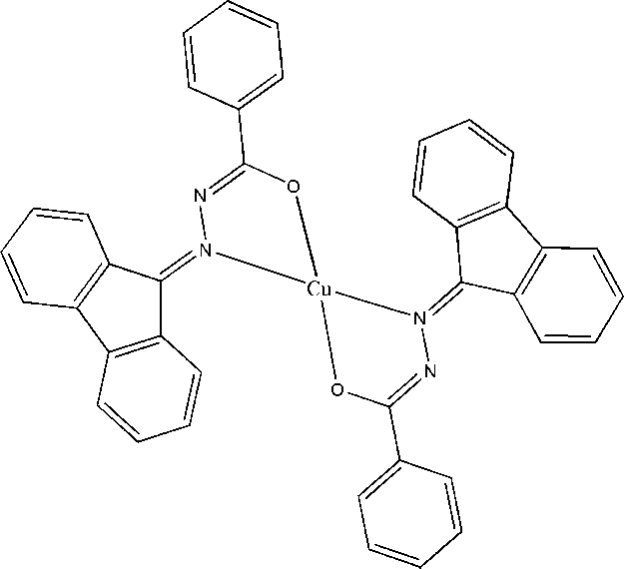


## Experimental

### 

#### Crystal data

[Cu(C_20_H_13_N_2_O)_2_]
*M* *_r_* = 658.19
Monoclinic, 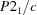

*a* = 14.437 (2) Å
*b* = 25.882 (3) Å
*c* = 8.1047 (2) Å
β = 98.205 (3)°
*V* = 2997.4 (6) Å^3^
*Z* = 4
Mo *K*α radiation
μ = 0.77 mm^−1^
*T* = 293 K
0.29 × 0.14 × 0.06 mm

#### Data collection

Bruker SMART CCD area-detector diffractometer
Absorption correction: multi-scan (*SADABS*; Sheldrick, 1996 ▶) *T*_min_ = 0.807, *T*_max_ = 0.955
15492 measured reflections
5331 independent reflections
2230 reflections with *I* > 2σ(*I*)
*R*_int_ = 0.131

#### Refinement

*R*[*F* ^2^ > 2σ(*F* ^2^)] = 0.070
*wR*(*F* ^2^) = 0.227
*S* = 1.02
5331 reflections
452 parameters
127 restraints
H-atom parameters constrained
Δρ_max_ = 0.59 e Å^−3^
Δρ_min_ = −0.70 e Å^−3^

### 

Data collection: *SMART* (Bruker, 1997) ▶; cell refinement: *SAINT* (Bruker, 1997) ▶; data reduction: *SAINT* ▶; program(s) used to solve structure: *SHELXS97* (Sheldrick, 2008 ▶); program(s) used to refine structure: *SHELXL97* (Sheldrick, 2008 ▶); molecular graphics: *PLATON* (Spek, 2009 ▶); software used to prepare material for publication: *SHELXTL* (Sheldrick, 2008 ▶).

## Supplementary Material

Crystal structure: contains datablock(s) I. DOI: 10.1107/S1600536811038931/bx2365sup1.cif

Structure factors: contains datablock(s) I. DOI: 10.1107/S1600536811038931/bx2365Isup2.hkl

Additional supplementary materials:  crystallographic information; 3D view; checkCIF report

## Figures and Tables

**Table 1:** Selected bond lengths (Å)

Cu1—O2	1.881 (5)
Cu1—O1	1.890 (5)
Cu1—N3	1.986 (6)
Cu1—N2	1.999 (6)

**Table 2:** Hydrogen-bond geometry (Å, °)

*D*—H⋯*A*	*D*—H	H⋯*A*	*D*⋯*A*	*D*—H⋯*A*
C3—H3⋯N1	0.93	2.37	2.888 (10)	115
C11—H11⋯O2	0.93	2.40	2.918 (10)	115
C23—H23⋯O1	0.93	2.39	3.007 (10)	124
C31—H31⋯N4	0.93	2.38	2.898 (9)	115

**Fig. 1.**
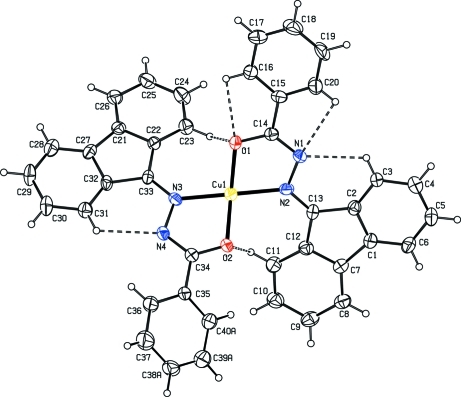
View of the title compound showing the atom-labelling scheme. Displacement ellipsoids are drawn at the 30% probability level. The hydrogen bonds are showed as dashed line.

## supplementary crystallographic information

### Comment

Aroylhydrazones have attracted much attention for many years because of their biological and pharmacological activities (Ranford *et al.*, 1998; Zhong *et al.*, 2007; Wang *et al.*, 2009; Li *et al.*, 2010), and their easy coordination with transition , lanthanide , and main group metals with versatile coordination modes also prompts the interests of inorganic chemists (El-Sherif, 2009; Yang *et al.*, 2006; Carcelli *et al.*, 1995). Our research group is interested in bidentate aroylhydrazone Schiff bases derived from 9*H*-fluoren-9-one. We report here the crystal structure of the title complex (I). The crystal structure of complex (I) is illustrated in Figure 1. Selected bond lengths are given in Table 1. The structure of Cu^II^ complex shows that the central Cu(II) ion is surrounded by two nitrogen atoms (N2 and N3) with Cu—N distances of 1.999 (6)Å and 1.986 (6) Å, and two oxygen atoms (O1 and O2) with Cu—O distances of 1.890 (5)Å and 1.881 (5) Å, forming a distorted rectangle-planar geometry. The ligands in complex (I) are in the enol form. The molecular structure is stabilized by three C—H···O and three C—H···N intramolecular hydrogen bonds, Table 2. The crystal packing is stabilized by van der Waals interactions.

### Experimental

A solution containing hydrated Cu(OAc)_2_ (0.15 mmol) and the ligand *N*'-(9*H*-fluoren-9-ylidene)benzohydrazide (0.30 mmol) in 15 cm of EtOH/DMF (2:1, v:v) was refluxed for 6 h. The precipitates thus produced were collected, washed several times with warm ethanol and dried *in vacuo*. Dark green single crystals suitable for X-ray diffraction analyses were obtained by slow evaporation of a solution of complex(I) in DMSO. Atoms C38,C39 & C40 are disordered and were modelled using a split model with refinend population parameters of 0.625(18 )/0.375 (18)

### Refinement

All H atoms were placed in geometrically idealized positions and constrained to ride on their parent atoms, with C—H = 0.93 Å, and with *U*_iso_(H) = 1.2*U*_eq_(C). Atoms C38, C39 & C40 are disordered and were modelled using a split model with refined population parameters of 0.625 (18)/0.375 (18).

### Crystal data

**Table:**

[Cu(C_20_H_13_N_2_O)_2_]	*F*(000) = 1356
*M**_r_* = 658.19	*D*_x_ = 1.459 Mg m^−^^3^
Monoclinic, *P*2_1_/*c*	Mo *K*α radiation, λ = 0.71073 Å
Hall symbol: -P 2ybc	Cell parameters from 1436 reflections
*a* = 14.437 (2) Å	θ = 2.8–18.4°
*b* = 25.882 (3) Å	µ = 0.77 mm^−^^1^
*c* = 8.1047 (2) Å	*T* = 293 K
β = 98.205 (3)°	Block, green
*V* = 2997.4 (6) Å^3^	0.29 × 0.14 × 0.06 mm
*Z* = 4	

### Data collection

**Table:**

Bruker SMART CCD area-detector diffractometer	5331 independent reflections
Radiation source: fine-focus sealed tube	2230 reflections with *I* > 2σ(*I*)
graphite	*R*_int_ = 0.131
phi and ω scans	θ_max_ = 25.1°, θ_min_ = 1.4°
Absorption correction: multi-scan (*SADABS*; Sheldrick, 1996)	*h* = −17→17
*T*_min_ = 0.807, *T*_max_ = 0.955	*k* = −30→25
15492 measured reflections	*l* = −9→9

### Refinement

**Table:**

Refinement on *F*^2^	Primary atom site location: structure-invariant direct methods
Least-squares matrix: full	Secondary atom site location: difference Fourier map
*R*[*F*^2^ > 2σ(*F*^2^)] = 0.070	Hydrogen site location: inferred from neighbouring sites
*wR*(*F*^2^) = 0.227	H-atom parameters constrained
*S* = 1.02	*w* = 1/[σ^2^(*F*_o_^2^) + (0.0807*P*)^2^ + 2.1941*P*] where *P* = (*F*_o_^2^ + 2*F*_c_^2^)/3
5331 reflections	(Δ/σ)_max_ < 0.001
452 parameters	Δρ_max_ = 0.59 e Å^−^^3^
127 restraints	Δρ_min_ = −0.70 e Å^−^^3^

### Special details

**Table:**

Refinement. Refinement of *F*^2^ against ALL reflections. The weighted *R*-factor *wR* and goodness of fit *S* are based on *F*^2^, conventional *R*-factors *R* are based on *F*, with *F* set to zero for negative *F*^2^. The threshold expression of *F*^2^ > σ(*F*^2^) is used only for calculating *R*-factors(gt) *etc*. and is not relevant to the choice of reflections for refinement. *R*-factors based on *F*^2^ are statistically about twice as large as those based on *F*, and *R*- factors based on ALL data will be even larger.

### Fractional atomic coordinates and isotropic or equivalent isotropic displacement parameters (Å^2^)

**Table:**

	*x*	*y*	*z*	*U*_iso_*/*U*_eq_	Occ. (<1)
C40B	−0.0047 (13)	0.3112 (6)	1.038 (4)	0.063 (6)	0.375 (18)
H40B	0.0586	0.3030	1.0559	0.075*	0.375 (18)
Cu1	0.20775 (6)	0.43559 (4)	0.93743 (12)	0.0450 (4)	
C1	0.4472 (6)	0.2777 (3)	1.0219 (10)	0.046 (2)	
C2	0.4509 (5)	0.3302 (3)	1.0635 (10)	0.042 (2)	
C3	0.5272 (5)	0.3499 (3)	1.1633 (11)	0.051 (2)	
H3	0.5300	0.3847	1.1921	0.062*	
C4	0.5998 (6)	0.3168 (4)	1.2201 (12)	0.065 (3)	
H4	0.6519	0.3297	1.2884	0.078*	
C5	0.5971 (6)	0.2657 (4)	1.1787 (13)	0.073 (3)	
H5	0.6470	0.2442	1.2185	0.087*	
C6	0.5202 (6)	0.2459 (3)	1.0774 (12)	0.059 (2)	
H6	0.5182	0.2112	1.0476	0.070*	
C7	0.3594 (6)	0.2663 (3)	0.9136 (10)	0.046 (2)	
C8	0.3236 (6)	0.2209 (3)	0.8465 (11)	0.054 (2)	
H8	0.3562	0.1902	0.8700	0.064*	
C9	0.2387 (7)	0.2209 (3)	0.7436 (11)	0.058 (2)	
H9	0.2129	0.1901	0.6998	0.070*	
C10	0.1914 (6)	0.2678 (3)	0.7055 (11)	0.059 (2)	
H10	0.1355	0.2681	0.6324	0.071*	
C11	0.2272 (6)	0.3133 (3)	0.7757 (10)	0.051 (2)	
H11	0.1957	0.3443	0.7503	0.062*	
C12	0.3096 (5)	0.3127 (3)	0.8833 (10)	0.045 (2)	
C13	0.3615 (5)	0.3537 (3)	0.9830 (9)	0.0403 (19)	
C14	0.3626 (5)	0.4804 (3)	1.0841 (9)	0.0383 (19)	
C15	0.4194 (5)	0.5219 (3)	1.1753 (10)	0.045 (2)	
C16	0.3900 (6)	0.5718 (3)	1.1627 (12)	0.063 (3)	
H16	0.3338	0.5800	1.0968	0.076*	
C17	0.4435 (7)	0.6108 (4)	1.2478 (14)	0.079 (3)	
H17	0.4229	0.6448	1.2383	0.095*	
C18	0.5245 (7)	0.5994 (4)	1.3434 (13)	0.074 (3)	
H18	0.5597	0.6257	1.4005	0.089*	
C19	0.5556 (6)	0.5496 (4)	1.3573 (14)	0.085 (3)	
H19	0.6111	0.5416	1.4258	0.102*	
C20	0.5039 (6)	0.5109 (3)	1.2684 (13)	0.071 (3)	
H20	0.5268	0.4773	1.2721	0.086*	
C21	0.0866 (5)	0.5813 (3)	0.5806 (9)	0.041 (2)	
C22	0.1356 (5)	0.5405 (3)	0.6667 (9)	0.0388 (19)	
C23	0.2302 (6)	0.5343 (3)	0.6566 (10)	0.051 (2)	
H23	0.2637	0.5074	0.7127	0.061*	
C24	0.2735 (6)	0.5684 (4)	0.5627 (11)	0.063 (2)	
H24	0.3371	0.5645	0.5577	0.076*	
C25	0.2258 (6)	0.6081 (4)	0.4760 (11)	0.064 (3)	
H25	0.2563	0.6301	0.4109	0.076*	
C26	0.1300 (6)	0.6151 (3)	0.4868 (11)	0.057 (2)	
H26	0.0969	0.6423	0.4311	0.069*	
C27	−0.0120 (6)	0.5776 (3)	0.6001 (10)	0.044 (2)	
C28	−0.0856 (6)	0.6088 (3)	0.5426 (11)	0.058 (2)	
H28	−0.0761	0.6386	0.4828	0.070*	
C29	−0.1738 (6)	0.5961 (4)	0.5736 (11)	0.064 (3)	
H29	−0.2241	0.6174	0.5352	0.077*	
C30	−0.1880 (6)	0.5519 (3)	0.6611 (11)	0.060 (3)	
H30	−0.2484	0.5433	0.6785	0.071*	
C31	−0.1143 (5)	0.5200 (3)	0.7240 (10)	0.047 (2)	
H31	−0.1241	0.4907	0.7859	0.056*	
C32	−0.0264 (5)	0.5330 (3)	0.6920 (9)	0.0391 (19)	
C33	0.0677 (5)	0.5100 (3)	0.7419 (9)	0.0358 (18)	
C34	0.0387 (5)	0.3958 (3)	0.9363 (10)	0.043 (2)	
C35	−0.0326 (5)	0.3590 (3)	0.9726 (10)	0.050 (2)	
C36	−0.1235 (5)	0.3734 (3)	0.9799 (11)	0.066 (3)	
H36	−0.1412	0.4077	0.9625	0.080*	
C37	−0.1880 (6)	0.3375 (3)	1.0124 (12)	0.093 (4)	
H37A	−0.2381	0.3466	1.0383	0.112*	0.50
H37B	−0.2481	0.3456	0.9943	0.112*	0.50
C38A	−0.1667 (8)	0.2850 (4)	1.003 (2)	0.066 (4)	0.625 (18)
H38A	−0.2112	0.2603	1.0206	0.079*	0.625 (18)
C39A	−0.0803 (9)	0.2695 (4)	0.967 (2)	0.070 (4)	0.625 (18)
H39A	−0.0677	0.2346	0.9530	0.084*	0.625 (18)
C40A	−0.0125 (9)	0.3066 (4)	0.953 (2)	0.063 (4)	0.625 (18)
H40A	0.0463	0.2967	0.9300	0.076*	0.625 (18)
C38B	−0.1612 (13)	0.2901 (6)	1.082 (4)	0.074 (6)	0.375 (18)
H38B	−0.2028	0.2690	1.1286	0.089*	0.375 (18)
C39B	−0.0692 (12)	0.2756 (6)	1.078 (4)	0.069 (5)	0.375 (18)
H39B	−0.0507	0.2417	1.1032	0.082*	0.375 (18)
N1	0.3951 (4)	0.4335 (2)	1.0919 (8)	0.0445 (16)	
N2	0.3319 (4)	0.4013 (2)	0.9993 (8)	0.0412 (16)	
N3	0.0894 (4)	0.4678 (2)	0.8305 (8)	0.0377 (15)	
N4	0.0121 (4)	0.4397 (2)	0.8676 (7)	0.0402 (15)	
O1	0.2795 (3)	0.49479 (18)	1.0070 (7)	0.0481 (15)	
O2	0.1258 (3)	0.38160 (19)	0.9738 (7)	0.0505 (15)	

### Atomic displacement parameters (Å^2^)

**Table:**

	*U*^11^	*U*^22^	*U*^33^	*U*^12^	*U*^13^	*U*^23^
C40B	0.061 (8)	0.056 (8)	0.070 (9)	0.002 (7)	0.003 (7)	0.013 (7)
Cu1	0.0386 (6)	0.0385 (6)	0.0539 (7)	0.0002 (5)	−0.0067 (5)	0.0015 (5)
C1	0.046 (5)	0.046 (5)	0.045 (5)	0.005 (4)	0.004 (4)	0.004 (4)
C2	0.041 (5)	0.044 (5)	0.043 (5)	0.006 (4)	0.011 (4)	0.004 (4)
C3	0.038 (5)	0.041 (5)	0.071 (7)	0.002 (4)	−0.006 (5)	0.003 (4)
C4	0.047 (6)	0.066 (7)	0.080 (7)	0.001 (5)	−0.003 (5)	0.005 (5)
C5	0.045 (6)	0.065 (7)	0.105 (9)	0.015 (5)	0.000 (6)	0.020 (6)
C6	0.054 (6)	0.042 (5)	0.081 (7)	0.012 (5)	0.013 (5)	0.004 (5)
C7	0.054 (5)	0.039 (5)	0.046 (5)	0.001 (4)	0.010 (4)	0.001 (4)
C8	0.059 (6)	0.044 (5)	0.061 (6)	−0.002 (4)	0.018 (5)	0.001 (4)
C9	0.077 (7)	0.045 (5)	0.053 (6)	−0.005 (5)	0.007 (5)	−0.009 (4)
C10	0.058 (6)	0.060 (6)	0.059 (6)	−0.005 (5)	0.005 (5)	−0.008 (5)
C11	0.060 (6)	0.048 (5)	0.043 (5)	0.003 (4)	−0.003 (5)	−0.006 (4)
C12	0.046 (5)	0.046 (5)	0.040 (5)	−0.003 (4)	0.002 (4)	−0.003 (4)
C13	0.039 (5)	0.042 (5)	0.040 (5)	0.009 (4)	0.004 (4)	0.004 (4)
C14	0.039 (5)	0.038 (5)	0.039 (5)	−0.002 (4)	0.011 (4)	−0.002 (4)
C15	0.043 (5)	0.041 (5)	0.052 (6)	−0.011 (4)	0.013 (4)	−0.008 (4)
C16	0.055 (6)	0.043 (6)	0.085 (7)	0.002 (4)	−0.018 (5)	−0.007 (5)
C17	0.072 (7)	0.048 (6)	0.113 (9)	−0.015 (5)	−0.004 (7)	−0.020 (6)
C18	0.061 (7)	0.069 (7)	0.092 (8)	−0.017 (6)	0.007 (6)	−0.034 (6)
C19	0.044 (6)	0.079 (8)	0.118 (10)	−0.001 (5)	−0.033 (6)	−0.029 (6)
C20	0.054 (6)	0.053 (6)	0.098 (8)	0.011 (5)	−0.020 (6)	−0.026 (5)
C21	0.045 (5)	0.039 (5)	0.038 (5)	−0.005 (4)	−0.002 (4)	−0.003 (4)
C22	0.039 (5)	0.037 (4)	0.037 (5)	0.000 (4)	−0.003 (4)	0.000 (4)
C23	0.047 (5)	0.055 (6)	0.049 (6)	0.002 (4)	0.003 (4)	0.002 (4)
C24	0.047 (6)	0.087 (7)	0.054 (6)	−0.009 (5)	0.001 (5)	0.008 (5)
C25	0.051 (6)	0.074 (7)	0.065 (7)	−0.016 (5)	0.003 (5)	0.009 (5)
C26	0.053 (6)	0.054 (6)	0.060 (6)	−0.008 (5)	−0.007 (5)	0.011 (5)
C27	0.057 (6)	0.034 (5)	0.038 (5)	0.007 (4)	−0.003 (4)	0.000 (4)
C28	0.066 (6)	0.052 (6)	0.055 (6)	0.012 (5)	0.002 (5)	0.006 (4)
C29	0.056 (6)	0.082 (7)	0.054 (6)	0.031 (5)	0.001 (5)	0.018 (5)
C30	0.053 (6)	0.070 (7)	0.052 (6)	0.024 (5)	−0.004 (5)	0.002 (5)
C31	0.044 (5)	0.053 (5)	0.044 (5)	0.005 (4)	0.013 (4)	0.002 (4)
C32	0.038 (5)	0.044 (5)	0.032 (5)	0.008 (4)	−0.008 (4)	−0.003 (4)
C33	0.037 (5)	0.035 (4)	0.033 (5)	0.009 (4)	−0.001 (4)	−0.002 (3)
C34	0.038 (5)	0.043 (5)	0.046 (5)	−0.001 (4)	0.003 (4)	0.001 (4)
C35	0.039 (5)	0.042 (5)	0.064 (6)	−0.004 (4)	−0.010 (4)	0.013 (4)
C36	0.044 (6)	0.064 (6)	0.094 (8)	−0.001 (5)	0.018 (5)	0.029 (5)
C37	0.061 (7)	0.096 (9)	0.126 (11)	−0.003 (6)	0.021 (7)	0.043 (7)
C38A	0.054 (6)	0.070 (7)	0.073 (8)	−0.014 (6)	0.011 (6)	0.021 (6)
C39A	0.071 (6)	0.064 (6)	0.071 (7)	−0.001 (5)	0.001 (6)	0.013 (6)
C40A	0.060 (7)	0.057 (7)	0.067 (8)	−0.003 (6)	−0.009 (6)	0.021 (6)
C38B	0.067 (8)	0.076 (8)	0.080 (9)	−0.011 (7)	0.013 (7)	0.012 (7)
C39B	0.064 (7)	0.063 (7)	0.076 (8)	0.004 (6)	0.001 (7)	0.020 (7)
N1	0.037 (4)	0.040 (4)	0.053 (4)	−0.003 (3)	−0.004 (3)	−0.007 (3)
N2	0.045 (4)	0.033 (4)	0.045 (4)	−0.008 (3)	0.003 (3)	−0.001 (3)
N3	0.030 (4)	0.041 (4)	0.042 (4)	−0.004 (3)	0.003 (3)	−0.005 (3)
N4	0.034 (4)	0.044 (4)	0.040 (4)	0.000 (3)	−0.002 (3)	0.003 (3)
O1	0.039 (3)	0.037 (3)	0.064 (4)	0.006 (2)	−0.008 (3)	0.001 (3)
O2	0.032 (3)	0.045 (3)	0.070 (4)	−0.003 (3)	−0.008 (3)	0.013 (3)

### Geometric parameters (Å, °)

**Table:**

C40B—C35	1.383 (10)	C21—C26	1.368 (10)
C40B—C39B	1.383 (9)	C21—C22	1.400 (10)
C40B—H40B	0.9300	C21—C27	1.457 (10)
Cu1—O2	1.881 (5)	C22—C23	1.388 (10)
Cu1—O1	1.890 (5)	C22—C33	1.460 (10)
Cu1—N3	1.986 (6)	C23—C24	1.372 (11)
Cu1—N2	1.999 (6)	C23—H23	0.9300
C1—C6	1.362 (10)	C24—C25	1.374 (11)
C1—C2	1.399 (10)	C24—H24	0.9300
C1—C7	1.466 (11)	C25—C26	1.409 (11)
C2—C3	1.367 (10)	C25—H25	0.9300
C2—C13	1.491 (10)	C26—H26	0.9300
C3—C4	1.381 (11)	C27—C28	1.364 (10)
C3—H3	0.9300	C27—C32	1.405 (10)
C4—C5	1.364 (11)	C28—C29	1.372 (11)
C4—H4	0.9300	C28—H28	0.9300
C5—C6	1.382 (12)	C29—C30	1.375 (11)
C5—H5	0.9300	C29—H29	0.9300
C6—H6	0.9300	C30—C31	1.387 (10)
C7—C8	1.365 (10)	C30—H30	0.9300
C7—C12	1.403 (10)	C31—C32	1.373 (10)
C8—C9	1.381 (11)	C31—H31	0.9300
C8—H8	0.9300	C32—C33	1.486 (9)
C9—C10	1.405 (11)	C33—N3	1.318 (8)
C9—H9	0.9300	C34—N4	1.299 (9)
C10—C11	1.377 (10)	C34—O2	1.303 (8)
C10—H10	0.9300	C34—C35	1.464 (9)
C11—C12	1.371 (10)	C35—C36	1.374 (7)
C11—H11	0.9300	C35—C40A	1.400 (9)
C12—C13	1.471 (10)	C36—C37	1.367 (7)
C13—N2	1.316 (8)	C36—H36	0.9300
C14—N1	1.300 (8)	C37—C38B	1.383 (9)
C14—O1	1.325 (8)	C37—C38A	1.398 (8)
C14—C15	1.481 (10)	C37—H37A	0.8168
C15—C16	1.360 (10)	C37—H37B	0.8852
C15—C20	1.370 (11)	C38A—C39A	1.380 (9)
C16—C17	1.390 (11)	C38A—H38A	0.9300
C16—H16	0.9300	C39A—C40A	1.389 (9)
C17—C18	1.341 (12)	C39A—H39A	0.9300
C17—H17	0.9300	C40A—H40A	0.9300
C18—C19	1.365 (12)	C38B—C39B	1.384 (9)
C18—H18	0.9300	C38B—H38B	0.9300
C19—C20	1.387 (11)	C39B—H39B	0.9300
C19—H19	0.9300	N1—N2	1.378 (8)
C20—H20	0.9300	N3—N4	1.400 (8)
			
C35—C40B—C39B	121.3 (12)	C23—C24—C25	121.9 (8)
C35—C40B—H40B	119.4	C23—C24—H24	119.0
C39B—C40B—H40B	119.4	C25—C24—H24	119.0
O2—Cu1—O1	152.3 (2)	C24—C25—C26	119.3 (8)
O2—Cu1—N3	81.9 (2)	C24—C25—H25	120.3
O1—Cu1—N3	101.0 (2)	C26—C25—H25	120.3
O2—Cu1—N2	101.1 (2)	C21—C26—C25	118.9 (8)
O1—Cu1—N2	81.5 (2)	C21—C26—H26	120.5
N3—Cu1—N2	168.8 (2)	C25—C26—H26	120.5
C6—C1—C2	120.4 (8)	C28—C27—C32	120.1 (8)
C6—C1—C7	129.7 (8)	C28—C27—C21	130.4 (8)
C2—C1—C7	109.8 (7)	C32—C27—C21	109.5 (7)
C3—C2—C1	120.3 (7)	C27—C28—C29	119.5 (8)
C3—C2—C13	132.9 (7)	C27—C28—H28	120.2
C1—C2—C13	106.8 (7)	C29—C28—H28	120.2
C2—C3—C4	118.4 (8)	C28—C29—C30	120.3 (8)
C2—C3—H3	120.8	C28—C29—H29	119.8
C4—C3—H3	120.8	C30—C29—H29	119.8
C5—C4—C3	121.7 (9)	C29—C30—C31	121.4 (8)
C5—C4—H4	119.2	C29—C30—H30	119.3
C3—C4—H4	119.2	C31—C30—H30	119.3
C4—C5—C6	119.9 (8)	C32—C31—C30	117.8 (8)
C4—C5—H5	120.0	C32—C31—H31	121.1
C6—C5—H5	120.0	C30—C31—H31	121.1
C1—C6—C5	119.3 (8)	C31—C32—C27	120.9 (7)
C1—C6—H6	120.3	C31—C32—C33	133.1 (7)
C5—C6—H6	120.3	C27—C32—C33	105.9 (7)
C8—C7—C12	120.8 (8)	N3—C33—C22	123.7 (7)
C8—C7—C1	131.1 (8)	N3—C33—C32	128.1 (7)
C12—C7—C1	108.1 (7)	C22—C33—C32	108.1 (6)
C7—C8—C9	119.5 (8)	N4—C34—O2	124.3 (7)
C7—C8—H8	120.2	N4—C34—C35	118.8 (7)
C9—C8—H8	120.2	O2—C34—C35	116.8 (7)
C8—C9—C10	119.7 (8)	C36—C35—C40B	116.9 (10)
C8—C9—H9	120.2	C36—C35—C40A	118.8 (8)
C10—C9—H9	120.2	C40B—C35—C40A	29.0 (12)
C11—C10—C9	120.4 (9)	C36—C35—C34	122.4 (7)
C11—C10—H10	119.8	C40B—C35—C34	119.1 (10)
C9—C10—H10	119.8	C40A—C35—C34	116.4 (8)
C12—C11—C10	119.6 (8)	C37—C36—C35	120.3 (7)
C12—C11—H11	120.2	C37—C36—H36	119.8
C10—C11—H11	120.2	C35—C36—H36	119.8
C11—C12—C7	119.8 (7)	C36—C37—C38B	121.6 (9)
C11—C12—C13	132.0 (7)	C36—C37—C38A	119.3 (8)
C7—C12—C13	108.2 (7)	C38B—C37—C38A	27.0 (13)
N2—C13—C12	125.6 (7)	C36—C37—H37A	120.5
N2—C13—C2	127.6 (7)	C38B—C37—H37A	110.9
C12—C13—C2	106.8 (7)	C38A—C37—H37A	120.2
N1—C14—O1	125.6 (7)	C36—C37—H37B	119.1
N1—C14—C15	118.8 (7)	C38B—C37—H37B	119.2
O1—C14—C15	115.5 (7)	C38A—C37—H37B	116.0
C16—C15—C20	118.7 (7)	H37A—C37—H37B	24.4
C16—C15—C14	120.5 (8)	C39A—C38A—C37	120.4 (9)
C20—C15—C14	120.7 (7)	C39A—C38A—H38A	119.8
C15—C16—C17	120.5 (9)	C37—C38A—H38A	119.8
C15—C16—H16	119.8	C38A—C39A—C40A	119.0 (10)
C17—C16—H16	119.8	C38A—C39A—H39A	120.5
C18—C17—C16	120.3 (9)	C40A—C39A—H39A	120.5
C18—C17—H17	119.8	C39A—C40A—C35	120.1 (10)
C16—C17—H17	119.8	C39A—C40A—H40A	120.0
C17—C18—C19	120.2 (9)	C35—C40A—H40A	120.0
C17—C18—H18	119.9	C37—C38B—C39B	116.5 (11)
C19—C18—H18	119.9	C37—C38B—H38B	121.8
C18—C19—C20	119.5 (9)	C39B—C38B—H38B	121.8
C18—C19—H19	120.2	C40B—C39B—C38B	120.3 (12)
C20—C19—H19	120.2	C40B—C39B—H39B	119.9
C15—C20—C19	120.6 (8)	C38B—C39B—H39B	119.9
C15—C20—H20	119.7	C14—N1—N2	109.4 (6)
C19—C20—H20	119.7	C13—N2—N1	115.0 (6)
C26—C21—C22	121.3 (8)	C13—N2—Cu1	133.1 (5)
C26—C21—C27	129.4 (7)	N1—N2—Cu1	111.6 (4)
C22—C21—C27	109.1 (7)	C33—N3—N4	114.4 (6)
C23—C22—C21	119.3 (7)	C33—N3—Cu1	135.0 (5)
C23—C22—C33	133.4 (7)	N4—N3—Cu1	110.5 (4)
C21—C22—C33	107.2 (7)	C34—N4—N3	110.5 (6)
C24—C23—C22	119.3 (8)	C14—O1—Cu1	109.5 (4)
C24—C23—H23	120.4	C34—O2—Cu1	111.1 (5)
C22—C23—H23	120.4		
			
C6—C1—C2—C3	−1.3 (12)	C23—C22—C33—N3	4.2 (14)
C7—C1—C2—C3	−179.6 (7)	C21—C22—C33—N3	178.9 (7)
C6—C1—C2—C13	179.7 (7)	C23—C22—C33—C32	−172.5 (8)
C7—C1—C2—C13	1.4 (9)	C21—C22—C33—C32	2.2 (8)
C1—C2—C3—C4	0.4 (12)	C31—C32—C33—N3	2.4 (14)
C13—C2—C3—C4	179.2 (8)	C27—C32—C33—N3	179.6 (7)
C2—C3—C4—C5	0.2 (14)	C31—C32—C33—C22	179.0 (8)
C3—C4—C5—C6	0.0 (15)	C27—C32—C33—C22	−3.8 (8)
C2—C1—C6—C5	1.5 (13)	C39B—C40B—C35—C36	13 (3)
C7—C1—C6—C5	179.4 (8)	C39B—C40B—C35—C40A	−89 (3)
C4—C5—C6—C1	−0.8 (14)	C39B—C40B—C35—C34	178.4 (19)
C6—C1—C7—C8	4.4 (15)	N4—C34—C35—C36	−18.0 (12)
C2—C1—C7—C8	−177.6 (8)	O2—C34—C35—C36	162.9 (7)
C6—C1—C7—C12	−176.1 (8)	N4—C34—C35—C40B	177.0 (15)
C2—C1—C7—C12	2.0 (9)	O2—C34—C35—C40B	−2.1 (17)
C12—C7—C8—C9	2.0 (12)	N4—C34—C35—C40A	144.2 (10)
C1—C7—C8—C9	−178.5 (8)	O2—C34—C35—C40A	−34.8 (12)
C7—C8—C9—C10	1.7 (13)	C40B—C35—C36—C37	−15.4 (15)
C8—C9—C10—C11	−2.8 (13)	C40A—C35—C36—C37	17.5 (12)
C9—C10—C11—C12	0.1 (13)	C34—C35—C36—C37	179.3 (8)
C10—C11—C12—C7	3.6 (12)	C35—C36—C37—C38B	17.6 (16)
C10—C11—C12—C13	−176.1 (8)	C35—C36—C37—C38A	−13.7 (10)
C8—C7—C12—C11	−4.7 (12)	C36—C37—C38A—C39A	2.5 (15)
C1—C7—C12—C11	175.7 (7)	C38B—C37—C38A—C39A	−100 (3)
C8—C7—C12—C13	175.1 (7)	C37—C38A—C39A—C40A	5(2)
C1—C7—C12—C13	−4.5 (9)	C38A—C39A—C40A—C35	−1(2)
C11—C12—C13—N2	6.7 (14)	C36—C35—C40A—C39A	−10.2 (18)
C7—C12—C13—N2	−173.1 (7)	C40B—C35—C40A—C39A	84 (2)
C11—C12—C13—C2	−174.9 (8)	C34—C35—C40A—C39A	−173.1 (12)
C7—C12—C13—C2	5.3 (9)	C36—C37—C38B—C39B	−16 (3)
C3—C2—C13—N2	−4.6 (14)	C38A—C37—C38B—C39B	78 (3)
C1—C2—C13—N2	174.3 (8)	C35—C40B—C39B—C38B	−12 (4)
C3—C2—C13—C12	177.1 (9)	C37—C38B—C39B—C40B	12 (4)
C1—C2—C13—C12	−4.1 (8)	O1—C14—N1—N2	3.4 (10)
N1—C14—C15—C16	176.4 (8)	C15—C14—N1—N2	179.9 (6)
O1—C14—C15—C16	−6.7 (11)	C12—C13—N2—N1	−176.6 (7)
N1—C14—C15—C20	−1.0 (12)	C2—C13—N2—N1	5.4 (11)
O1—C14—C15—C20	175.8 (8)	C12—C13—N2—Cu1	11.1 (12)
C20—C15—C16—C17	−2.2 (14)	C2—C13—N2—Cu1	−167.0 (6)
C14—C15—C16—C17	−179.7 (9)	C14—N1—N2—C13	173.0 (6)
C15—C16—C17—C18	−0.1 (16)	C14—N1—N2—Cu1	−13.0 (7)
C16—C17—C18—C19	0.4 (17)	O2—Cu1—N2—C13	34.6 (7)
C17—C18—C19—C20	1.6 (17)	O1—Cu1—N2—C13	−173.3 (7)
C16—C15—C20—C19	4.3 (15)	N3—Cu1—N2—C13	−69.7 (15)
C14—C15—C20—C19	−178.2 (9)	O2—Cu1—N2—N1	−137.9 (5)
C18—C19—C20—C15	−4.0 (17)	O1—Cu1—N2—N1	14.2 (5)
C26—C21—C22—C23	0.0 (11)	N3—Cu1—N2—N1	117.7 (12)
C27—C21—C22—C23	175.8 (7)	C22—C33—N3—N4	−169.9 (6)
C26—C21—C22—C33	−175.5 (7)	C32—C33—N3—N4	6.2 (11)
C27—C21—C22—C33	0.3 (8)	C22—C33—N3—Cu1	14.2 (12)
C21—C22—C23—C24	0.1 (12)	C32—C33—N3—Cu1	−169.7 (5)
C33—C22—C23—C24	174.3 (8)	O2—Cu1—N3—C33	−172.5 (7)
C22—C23—C24—C25	−1.1 (13)	O1—Cu1—N3—C33	35.4 (7)
C23—C24—C25—C26	1.7 (14)	N2—Cu1—N3—C33	−66.2 (15)
C22—C21—C26—C25	0.6 (12)	O2—Cu1—N3—N4	11.5 (4)
C27—C21—C26—C25	−174.2 (8)	O1—Cu1—N3—N4	−140.6 (4)
C24—C25—C26—C21	−1.5 (13)	N2—Cu1—N3—N4	117.7 (12)
C26—C21—C27—C28	−6.3 (14)	O2—C34—N4—N3	3.7 (11)
C22—C21—C27—C28	178.4 (8)	C35—C34—N4—N3	−175.3 (6)
C26—C21—C27—C32	172.6 (8)	C33—N3—N4—C34	172.0 (7)
C22—C21—C27—C32	−2.8 (9)	Cu1—N3—N4—C34	−11.1 (7)
C32—C27—C28—C29	−0.7 (12)	N1—C14—O1—Cu1	8.5 (9)
C21—C27—C28—C29	178.0 (8)	C15—C14—O1—Cu1	−168.1 (5)
C27—C28—C29—C30	−0.4 (14)	O2—Cu1—O1—C14	86.0 (6)
C28—C29—C30—C31	1.7 (14)	N3—Cu1—O1—C14	179.5 (5)
C29—C30—C31—C32	−1.8 (13)	N2—Cu1—O1—C14	−11.6 (5)
C30—C31—C32—C27	0.7 (11)	N4—C34—O2—Cu1	6.2 (10)
C30—C31—C32—C33	177.6 (8)	C35—C34—O2—Cu1	−174.8 (6)
C28—C27—C32—C31	0.6 (11)	O1—Cu1—O2—C34	88.7 (7)
C21—C27—C32—C31	−178.4 (7)	N3—Cu1—O2—C34	−9.5 (5)
C28—C27—C32—C33	−177.1 (7)	N2—Cu1—O2—C34	−178.5 (5)
C21—C27—C32—C33	4.0 (8)		

### Hydrogen-bond geometry (Å, °)

**Table:**

*D*—H···*A*	*D*—H	H···*A*	*D*···*A*	*D*—H···*A*
C3—H3···N1	0.93	2.37	2.888 (10)	115
C11—H11···O2	0.93	2.40	2.918 (10)	115
C23—H23···O1	0.93	2.39	3.007 (10)	124
C31—H31···N4	0.93	2.38	2.898 (9)	115
